# Supplementary material for: From sequence to enzyme mechanism using multi-label machine learning
Source: BMC Bioinformatics. 2014 May 19;15:150. doi: 10.1186/1471-2105-15-150 (PMC4229970; doi:10.1186/1471-2105-15-150)
Supplement: Additional file 2 — Java code of ml2db. Additional file ml2db_code.tar.gz contains the Java source code to run the multi-label machine learning experiments and save the results to database. The code’s Javadoc is included. [file 1471-2105-15-150-S2.zip › additional file 2/ml2db/ml2db/doc/mulan/classifier/neural/package-summary.html]

mulan.classifier.neural


JavaScript is disabled on your browser.


- Overview
- Package
- Class
- Use
- Tree
- Deprecated
- Index
- Help

- Prev Package
- Next Package

- Frames
- No Frames

- All Classes

# Package mulan.classifier.neural

- Interface Summary

  | Interface | Description |
  | --- | --- |
  |  |  |
  | --- | --- |
  | ModelUpdateRule | Represents an update rule, which can be used by a learner, to process an input example in learning phase and perform an update of a model when necessary. |
- Class Summary

  | Class | Description |
  | --- | --- |
  |  |  |
  | --- | --- |
  | BPMLL | The implementation of Back-Propagation Multi-Label Learning (BPMLL) learner. |
  | BPMLLAlgorithm | The implementation of Back-Propagation Multi-Label Learning (BPMLL) algorithm for neural networks. |
  | DataPair | Class for representation of a data-pair instance. |
  | MMPLearner | Implementation of Multiclass Multilabel Perceptrons learner. |
  | MMPMaxUpdateRule | Implementation of max update rule for `MMPLearner`. |
  | MMPRandomizedUpdateRule | Implementation of randomized update rule for `MMPLearner`. |
  | MMPUniformUpdateRule | Implementation of uniform update rule for `MMPLearner`. |
  | MMPUpdateRuleBase | The base class of update rules for `MMPLearner`. |
  | NormalizationFilter | Performs a normalization of numeric attributes of the data set. |
  | ThresholdFunction | Implementation of a threshold function. |
- Enum Summary

  | Enum | Description |
  | --- | --- |
  |  |  |
  | --- | --- |
  | MMPUpdateRuleType | The enumeration of update rules, which can be used by the `MMPLearner` to update its model in learning phase. |

- Overview
- Package
- Class
- Use
- Tree
- Deprecated
- Index
- Help

- Prev Package
- Next Package

- Frames
- No Frames

- All Classes
